# Transitory Activation and Improved Transition from Erosion to Formation within Intracortical Bone Remodeling in Hypoparathyroid Patients Treated with rhPTH(1–84)

**DOI:** 10.1002/jbm4.10829

**Published:** 2023-11-17

**Authors:** Pernille van Dijk Christiansen, Tanja Sikjær, Christina Møller Andreasen, Jesper Skovhus Thomsen, Annemarie Brüel, Ellen Margrethe Hauge, Jean‐Marie Delaisse, Lars Rejnmark, Thomas Levin Andersen

**Affiliations:** ^1^ Department of Pathology Odense University Hospital Odense Denmark; ^2^ Molecular Bone Histology (MBH) Lab, Research Unit of Pathology, Department of Clinical Research and Department of Molecular Medicine University of Southern Denmark Odense Denmark; ^3^ Department of Endocrinology and Internal Medicine Aarhus University Hospital Aarhus Denmark; ^4^ Department of Clinical Medicine Aarhus University Aarhus Denmark; ^5^ Department of Biomedicine Aarhus University Aarhus Denmark; ^6^ Department of Rheumatology Aarhus University Hospital Aarhus Denmark; ^7^ Molecular Bone Histology (MBH) Lab, Department of Forensic Medicine Aarhus University Aarhus Denmark

**Keywords:** BONE FORMATION, BONE TURNOVER, HYPOPARATHYROIDISM, PARATHYROID HORMONE, RESORPTION‐REVERSAL PHASE, INTRACORTICAL BONE

## Abstract

In hypoparathyroidism, lack of parathyroid hormone (PTH) leads to low calcium levels and decreased bone remodeling. Treatment with recombinant human PTH (rhPTH) may normalize bone turnover. This study aimed to investigate whether rhPTH(1–84) continued to activate intracortical bone remodeling after 30 months and promoted the transition from erosion to formation and whether this effect was transitory when rhPTH(1–84) was discontinued. Cortical histomorphometry was performed on 60 bone biopsies from patients (aged 31 to 78 years) with chronic hypoparathyroidism randomized to either 100 μg rhPTH(1–84) a day (*n* = 21) (PTH) or similar placebo (*n* = 21) (PLB) for 6 months as add‐on to conventional therapy. This was followed by an open‐label extension, where patients extended their rhPTH(1–84) (PTH) (*n* = 5), continued conventional treatment (CON) (*n* = 5), or withdrew from rhPTH(1–84) and resumed conventional therapy (PTHw) for an additional 24 months (*n* = 8). Bone biopsies were collected at months 6 (*n* = 42) and 30 (*n* = 18). After 6 and 30 months, the overall cortical microarchitecture (cortical porosity, thickness, pore density, and mean pore diameter) in the PTH group did not differ from that of the PLB/CON and PTHw groups. Still, the PTH group had a significantly and persistently higher percentage of pores undergoing remodeling than the PLB/CON groups. A significantly higher percentage of these pores was undergoing bone formation in the PTH compared with the PLB/CON groups, whereas the percentage of pores with erosion only was not different. This resulted in a shift in the ratio between formative and eroded pores, reflecting a faster transition from erosion to formation in the PTH‐treated patients. In the rhPTH(1–84) withdrawal group PTHw, the latter effects of PTH were completely reversed in comparison to those of the PLB/CON groups. In conclusion, rhPTH(1–84) replacement therapy in hypoparathyroidism patients promotes intracortical remodeling and its transition from erosion to formation without affecting the overall cortical microstructure. The effect persists for at least 30 months and is reversible when treatment is withdrawn. © 2023 The Authors. *JBMR Plus* published by Wiley Periodicals LLC on behalf of American Society for Bone and Mineral Research.

## Graphical abstract

The present histomorphometric study aimed to investigate whether rhPTH(1–84) continued to activate intracortical bone remodeling after 30 months, was transitory when discontinued, and promoted the transition from erosion to formation in hypoparathyroidism patients. The study shows that rhPTH(1–84) replacement therapy in hypoparathyroidism patients promotes intracortical remodeling and its transition from erosion to formation without affecting the overall cortical microstructure. The effect persists for at least 30 months and is reversible when treatment is withdrawn.

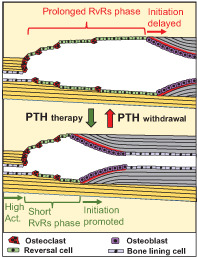

## Introduction

Parathyroid hormone (PTH) is responsible for regulating serum levels of calcium in the body.^[^ ^1^ ^]^ Low calcium levels trigger the release of PTH from the parathyroid glands. PTH increases serum calcium levels by stimulating calcium uptake in the gastrointestinal tract, calcium reabsorption in the kidneys, and release of calcium from the bones.^[^ ^2^, ^3^, ^4^, ^5^ ^]^ Hypoparathyroidism is a rare hormonal disease characterized by insufficient PTH production, resulting in an inadequate serum calcium concentration. The most prominent clinical symptom of hypoparathyroidism is neuromuscular irritability.^[^ ^6^ ^]^ Furthermore, patients with hypoparathyroidism have increased bone mineral density and reduced levels of bone turnover markers, corresponding to a low bone turnover.^[^ ^7^, ^8^, ^9^ ^]^

Conventional therapy for hypoparathyroidism is calcium supplements in combination with active vitamin D. Subcutaneous injections with recombinant human PTH (rhPTH[1–84]) are approved as PTH replacement therapy in patients with hypoparathyroidism who are not adequately controlled on conventional therapy.^[^ ^10^ ^]^ Even though rhPTH(1–84) has a longer half‐life than the truncated PTH(1–34) when used in patients with osteoporosis, its half‐life remains short. Accordingly, once‐a‐day injections with rhPTH(1–84) result in rapid, temporary bursts in serum PTH levels, in contrast to the physiological effects of endogenous PTH that are sustained more constantly.^[^ ^11^ ^]^ From a bone remodeling perspective, intermittent increases in PTH levels are considered to result in a more potent anabolic response than constantly elevated endogenous PTH levels, as seen in patients with hyperparathyroidism, but both result in an elevated bone turnover.^[^ ^3^, ^12^ ^]^ Whether the elevated bone turnover and anabolic response to intermittent PTH persist in long‐term treated patients (≥24 months) remains contradictory^[^ ^13^, ^14^, ^15^, ^16^ ^]^ and are therefore part of the focus of this study.

The bone remodeling process replaces old or damaged bone with new bone. This process is continuously ongoing in order to keep the skeleton strong and resistant to fractures.^[^ ^17^, ^18^ ^]^ Remodeling begins with osteoclastic bone resorption and ends with osteoblastic bone formation refilling the resorbed cavity. Bone resorption and formation are coupled by the intermediate reversal–resorption phase, where bone‐resorbing osteoclasts are intermixed with osteoblastic reversal cells on the eroded surfaces.^[^ ^19^, ^20^, ^21^, ^22^, ^23^, ^24^, ^25^ ^]^ Osteoblastic reversal cells are identical to osteoprogenitors that gradually differentiate into mature bone‐forming osteoblasts.^[^ ^19^, ^20^, ^22^ ^]^ Bone formation is initiated once these osteoprogenitors have colonized the eroded surfaces and reached a certain critical density obtained by recruitment and proliferation of these osteoprogenitors.^[^ ^19^, ^20^ ^]^ Importantly, bone loss during aging and osteoporosis has been shown to be the result of a delayed transition from bone erosion to formation, likely due to an insufficient expansion of the osteoprogenitor population on eroded surfaces.^[^ ^19^, ^20^, ^26^, ^27^, ^28^ ^]^ This results in a prolonged reversal–resorption phase and a delayed initiation of the subsequent formation phase. Whether anabolic therapies like intermittent PTH treatment promote the transition from erosion to formation within active remodeling events, rescuing the reported pathophysiology of aging and osteoporosis, remains an open question.

PTH binds the PTH 1 receptor (PTH1R) on the membrane of osteoblast‐lineage cells,^[^ ^2^, ^3^, ^4^, ^5^ ^]^ where it directly promotes osteoblastogenesis and bone formation.^[^ ^13^, ^29^, ^30^, ^31^ ^]^ In addition, PTH has an osteoblast‐mediated indirect effect on osteoclasts through the receptor activator of NF‐κB (RANK)/receptor activator of NF‐κB ligand (RANKL)/osteoprotegerin (OPG) pathway,^[^ ^32^ ^]^ promoting their activation and resorptive activity.^[^ ^33^, ^34^, ^35^ ^]^ Accordingly, both markers of bone resorption^[^ ^13^, ^14^, ^15^, ^36^, ^37^, ^38^, ^39^, ^40^, ^41^, ^42^, ^43^, ^44^, ^45^ ^]^ and formation^[^ ^31^, ^37^, ^40^, ^41^, ^42^, ^43^, ^44^, ^46^, ^47^, ^48^, ^49^ ^]^ increase following PTH treatment. However, the anabolic effect of PTH may not be as pronounced in cortical as in trabecular bone.^[^ ^2^ ^]^ Indeed, PTH has been reported to be less effective against fractures of non‐vertebral bones, mainly composed of cortical bone, than of fractures in vertebral bones, mainly composed of trabecular bone.^[^ ^50^ ^]^ Moreover, previous studies found no effect of PTH therapy on cortical porosity, pore density, or cortical thickness in osteoporotic patients,^[^ ^29^, ^30^, ^51^, ^52^ ^]^ further supporting the notion that PTH is less effective in cortical bone, even though some positive cortical effects have been reported.^[^ ^52^, ^53^ ^]^ Therefore, further studies are needed to clarify how PTH treatment affects cortical bone remodeling and its influence on the overall cortical bone microstructure.

This histomorphometric study investigated the effect of intermittent rhPTH(1–84) replacement therapy (i.e., once‐a‐day injections) on cortical bone microstructure and intracortical bone remodeling on patients with hypoparathyroidism. The study was performed on bone biopsies obtained from a 6‐month randomized, double‐blinded, placebo‐controlled study (rhPTH[1–84] and placebo groups), followed by a 24‐month open‐label study with three groups (30‐month rhPTH[1–84], 30‐month control, and 6‐month rhPTH[1–84] followed by 24‐month withdrawal).^[^ ^11^, ^31^ ^]^ Our aims were to investigate whether rhPTH(1–84) persistently activated bone remodeling for 30 months and promoted the transition from bone erosion to formation (coupling) in intracortical remodeling events and whether these effects were transitory when rhPTH(1–84) treatment was discontinued after 6 months. The study utilized a recently developed detailed intracortical histomorphometric analysis, which has been used to investigate the remodeling steps critical for age‐related cortical bone loss and cortical trabecularization,^[^ ^19^, ^53^, ^54^ ^]^ as well as for elucidating the intracortical effect of alendronate.^[^ ^55^ ^]^

## Materials and Methods

### Study participants and bone biopsy specimens

**Fig. 1:**
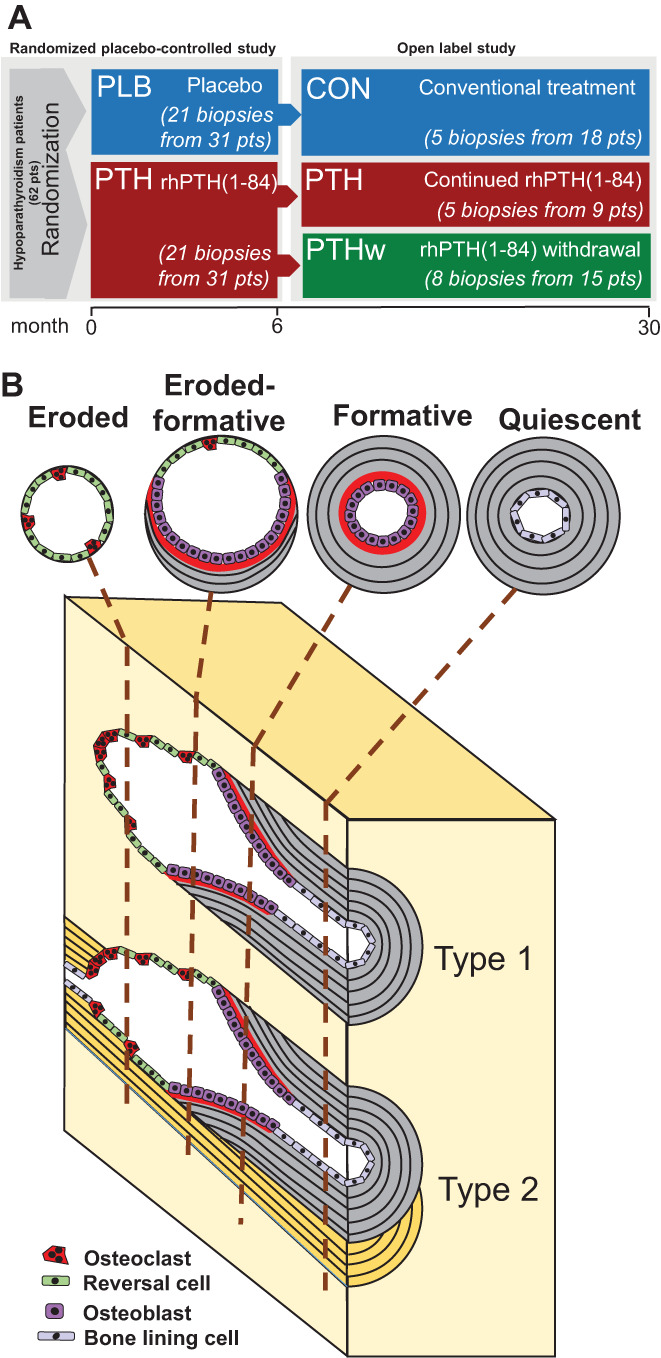
Study design and histomophometric classification of intracortical remodeling events. (A) The treatment groups include 6 months of treatment with placebo (PLB) or rhPTH(1–84) (PTH) and 30 months of treatment with control (CON) group (6 months of placebo followed by standard treatment for 24 months) and PTH or PTH withdrawal (PTHw) groups (6 months of PTH followed by standard treatment for 24 months). (B) Cortical histomorphometry investigates the remodeling type and stage of the intracortical remodeling, as illustrated in model of human intracortical remodeling events. Pores were classified according to their remodeling type to determine whether they generated a new pore/canal (type 1 remodeling) or remodeled an existing pore/canal (type 2 remodeling), as well as their remodeling stage. Here, the pores were classified as quiescent (no ongoing remodeling) or non‐quiescent (active) pores, including eroded, eroded‐formative, and formative pores.

This histomorphometric study was conducted using a total of 60 transiliac bone biopsies from 47 patients with chronic hypoparathyroidism (aged 31–78 years) randomized to either 100 μg rhPTH(1–84) a day (*n* = 21) (PTH) or similar placebo (*n* = 21) (PLB) for 6 months as add‐on to conventional therapy. The study was followed by an open‐label extension, in which patients continued their rhPTH(1–84) (PTH; *n* = 5), continued conventional treatment (CON; *n* = 5), or withdrew from rhPTH(1–84) and resumed conventional therapy for an additional 24 months (PTHw; *n* = 8). Bone biopsies were collected at month 6 (*n* = 42) and 30 (*n* = 18). It is important to note that five of the patients only had biopsies included at the 30‐month time point due to the quality of the cortical bone at month 6 (Fig. 1A). Transiliac crest bone biopsies, with a diameter of 6 to 8 mm, were obtained 2 cm behind and 2 cm below the anterior superior iliac spine. These biopsies reflected a subgroup of the patients included in the 6‐month, double‐blinded, place‐controlled clinical study and in the 24‐month open‐label extension (Fig. 1A). The bone biopsies were fixed in 70% ethanol, dehydrated, and embedded in methylmethacrylate. Note that the study originally included 71 biopsies from 52 patients, but 11 of these biopsies were not suitable for the cortical histomorphometry and therefore excluded. Therefore, five of the 30‐month biopsies lacked paired 6‐month biopsies.

As previously described, the overall clinical study included 62 patients (nine men and 53 women) aged 31 to 78 years with chronic hypoparathyroidism.^[^ ^11^, ^31^ ^]^ This biopsy‐based study, however, only included 47 patients (Fig. 1A). Of these 47 patients, 45 patients developed hypoparathyroidism following surgery from atoxic goiter (*n* = 18), toxic goiter (*n* = 13), thyroid cancer (*n* = 8), or primary hyperparathyroidism (*n* = 3). The remaining two patients had non‐surgical (idiopathic) hypoparathyroidism. The mean time from diagnosis to initiation of the study was 9 years, with a range of 1 to 37 years. Details regarding the age, gender, time since diagnosis, and etiology distribution of the patients included in each group of this biopsy study can be found in Supplementary Table S1. The inclusion and exclusion criteria were previously detailed.^[^ ^31^ ^]^ Patients were randomized to 6 months of therapy with either 100 μg rhPTH(1–84) (Preotact, Nycomed, Zürich, Switzerland) (PTH group) or placebo administered as a subcutaneous injections in the thigh once daily added to conventional therapy with oral active vitamin D analogs and calcium supplements (PLB group). If plasma calcium levels fell outside the reference range, the dosage of calcium and active vitamin D were adjusted according to a predefined scheme.^[^ ^31^ ^]^ The randomized study was followed by an open‐label extension for an additional 24 months. Some of the rhPTH(1–84)‐treated patients continued their PTH treatment (PTH group, *n* = 9 patients, *n* = 5 biopsies) or crossed over to conventional treatment (PTH withdrawal [PTHw] group, *n* = 15 patients, *n* = 8 biopsies), while the placebo‐treated patients continued with conventional treatment (CON group, *n* = 18 patients, *n* = 5 biopsies) (Fig. 1A).

The study was monitored by the Good Clinical Practice (GCP) Unit at Aarhus University Hospital, Denmark, and was performed in accordance with the Declaration of Helsinki II. The trial was registered as EudraCT No. 2008‐000606‐36 (sponsor protocol 84421383) and as ClinicalTrials.gov No. NCT00730210, with approval obtained from the Biomedical Research Ethics Committee of Central Denmark (M‐20080040).

### Specimen preparation for cortical histomorphometry

To prepare for the cortical histomorphometry, 7‐μm‐thick sections were cut using a Leica Jung RM 2065 microtome and stained with Masson‐Goldner trichrome. In brief, the plastic was removed with 2‐methoxymethylacetate before the sections were rehydrated. The tissue was stained with a mixture of Biebrich Scarlet and fuchsine acid, treated with phosphotungstic acid, and stained with Light Green. The nuclei were stained with Weigerts' hematoxylin. Finally, the sections were dehydrated and mounted with Pertex. Prior to histomorphometry, images of the stained sections were acquired using a Nanozoomer S360 digital slide scanner (Hamamatsu, Hamamatsu City, Japan).

### Cortical histomorphometric analysis

A single Masson‐Goldner trichrome‐stained section from each biopsy underwent a comprehensive cortical histomorphometric analysis using a combination of polarized light microscopy and measurements on the digital scans. During the histomorphometric analyses, the investigators were blinded for the group distribution.

First the cortical microstructure was investigated. The mean cortical thickness, cortical porosity, cortical pore density, and mean cortical pore diameter were determined as previously described.^[^ ^23^ ^]^ The measurements were performed on digital scans using Hamamatsu NDP.view2 software (Hamamatsu City, Japan).

Second, the intracortical pores were individually investigated and given an identification number across both entire cortexes within the digital scans using the NDP.view2 software. The analysis was carried out by two independent observers (PC and TS) and validated by two senior observers (CMA and TLA), ensuring that the pores were correctly measured and classified, as previously described.^[^ ^23^ ^]^ The measurements were performed on the digital scans assisted by polarized light microscopy to visualize the detailed lamellar structure of the cortical osteons. The pore diameter and area were determined for each of the intracortical pores, and the osteon diameter was determined, and then the wall thickness was derived for each of the quiescent pores/osteons. The pores were classified according to their remodeling type and stage:

1. Remodeling type, i.e., whether the pores reflected type 1 remodeling (generation of new pores) or type 2 remodeling (remodeling of existing pores);
2. Remodeling stage, i.e., whether the pore surface was quiescent (Q), eroded (E), mixed eroded and formative (EF), or formative (F). Remodeling stage E, EF, and F are collectively referred to as non‐quiescent pores.

Using this classification, the percentage of pores and their contribution to the total pore area of each remodeling type and stage was calculated for each patient.

### Statistical analysis

The relationship between porosity and pore diameter or density was examined using linear regression analysis. All data were tested for normal distribution using Shapiro–Wilk normality tests. At 6 months, statistically significant differences between the PLB and PTH groups were identified using *t* tests when the requirements for normal distribution were met; otherwise Mann–Whitney tests were used. At 30 months, statistically significant differences between the groups were calculated using one‐way ANOVA tests followed by Tukey's multiple comparison tests when the data passed the normality test; otherwise, Kruskal–Wallis tests followed by Dunn's multiple comparison tests were used. Statistically significant differences between 6 months of PTH compared to 30 months of PTH and PTHw were calculated using one‐way ANOVA tests followed by Dunnett's multiple comparison tests when the data passed the normality test; otherwise Kruskal–Wallis tests followed by Dunn's multiple comparison tests were used. Post hoc tests were only performed when the Kruskal–Wallis test or ANOVA had significant H‐ or F‐values. In addition, the paired biopsies at 6 months (PTH) and 30 months (PTH and PTHw) were compared using a paired *t* test. In all graphs, both the unpaired and paired analysis between 6 months (PTH) and 30 months (PTH and PTHw) are reported. All *p* values <0.1 were noted, and *p* values ≤0.05 were considered statistically significant. Statistical analyses were performed using GraphPad Prism version 8 (GraphPad Software Inc., La Jolla, CA, USA).

## Results

### Effect of rhPTH(1–84) on cortical microstructure

**Fig. 2:**
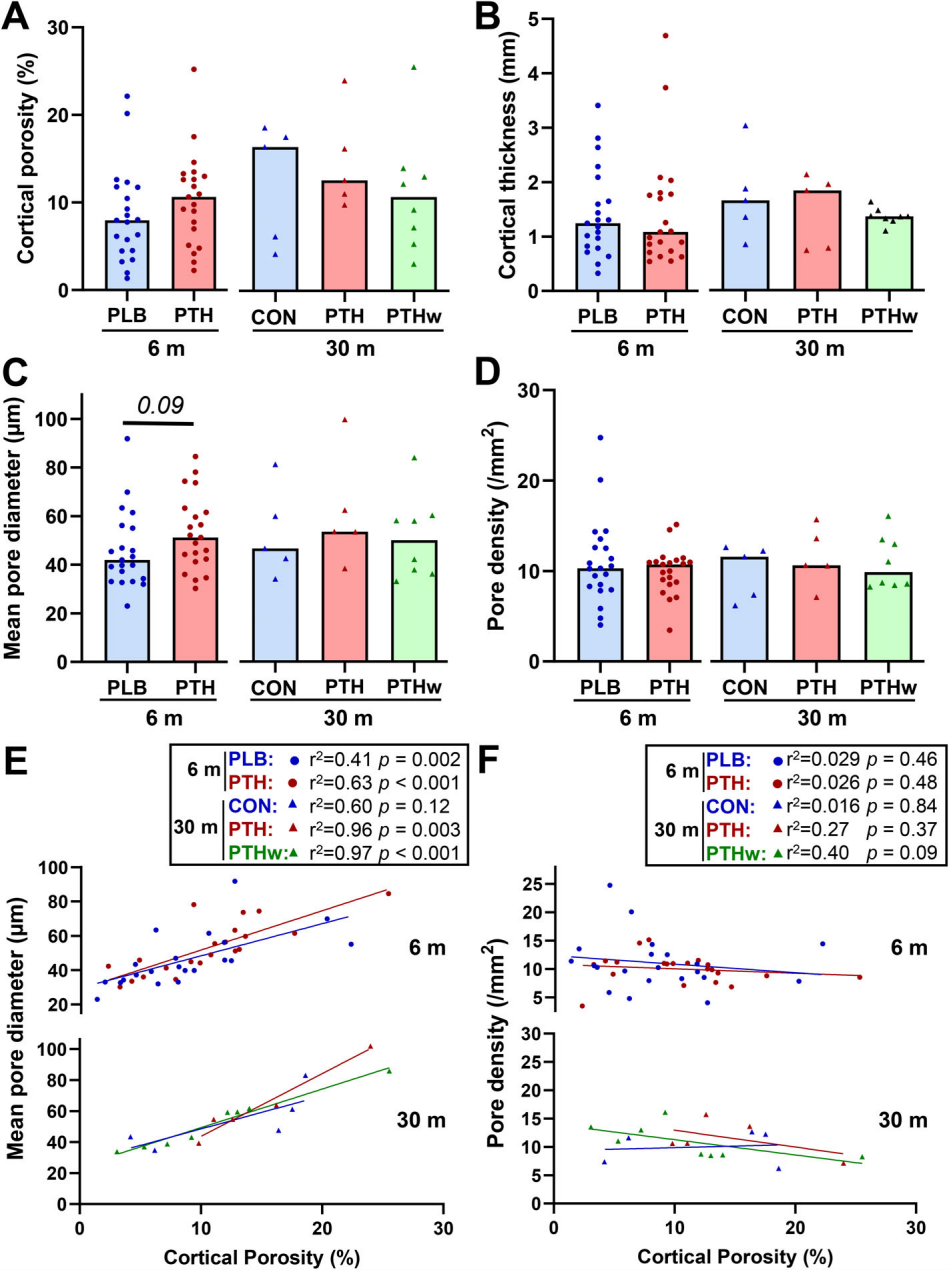
Effect of rhPTH(1–84) on overall cortical microstructure. (A–D) The study showed no significant effect on cortical porosity (A), cortical thickness (B), mean cortical pore diameter (C), or cortical pore density (D) within the respective treatment groups. Each dot represents a patient, and the bar represents the median. Normality was tested using the Shapiro–Wilk normality test. At 6 months, PLB and PTH were compared using *t* tests (A, B, D) or a Mann–Whitney test (C). At 30 months, CON, PTH, and PTHw were compared using one‐way ANOVA tests followed by Tukey's multiple comparison tests. Differences between PTH at 6 months and PTH and PTHw at 30 months were determined using one‐way ANOVA tests followed by Dunnett's multiple comparison tests. *p* values <0.1 are noted. (E–F) The cortical porosity was mainly driven by changes in the mean pore diameter rather than by changes in the pore density, as shown in the correlation between cortical porosity and mean pore diameter (E) or pore density (F). The correlations were calculated for all five groups using linear regressions, and the obtained fit lines are shown in the scatter plot, where each dot represents a patient.

The mean (across all groups) pore diameter (51 ± 17 μm), cortical porosity (10 ± 5.8%), cortical thickness (1.5 ± 0.82 mm), and pore density (11 ± 3.5 pores/mm^2^) did not differ between PTH and PLB/CON‐treated patients after either 6 or 30 months or in the PTHw group (Fig. 2). Linear regression analysis revealed that the cortical porosity was significantly correlated with pore diameter (Fig. 2E), but not with pore density (Fig. 2F), supporting the view that changes in cortical porosity are primarily driven by changes in pore size (Fig. 2E), rather than changes in pore density (Fig. 2F).

### Effect of rhPTH(1–84) on activation of intracortical bone remodeling

**Fig. 3:**
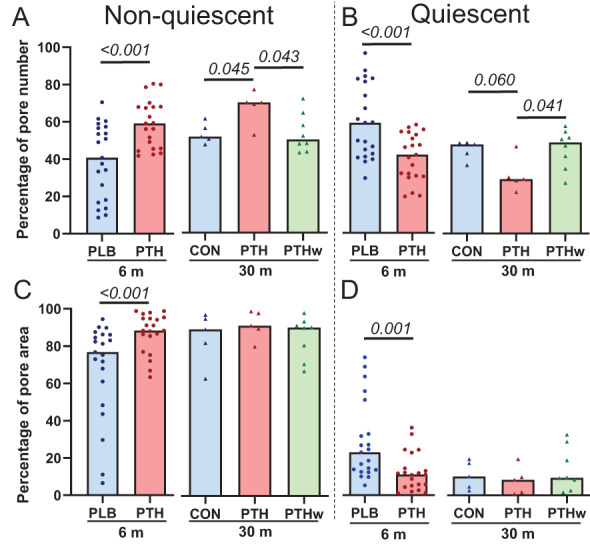
Effect of rhPTH(1–84) on non‐quiescent (active) and quiescent pores. (A, B) Percentages of non‐quiescent (active) cortical pores significantly increased in the PTH groups after 6 and 30 months compared to the PLB/CON groups (A), while the opposite was the case for quiescent cortical pores (B). The PTHw normalized to the levels of the PLB/CON groups for both non‐quiescent and quiescent pores. (C, D) At 6 months, the PTH group showed a significant increase in the contribution of non‐quiescent pores to the total pore area (C), while the opposite was true for quiecent pores (D). At 30 months, there were no significant differences between the groups. Each dot represents a patient, and the bar represents the median. Normality was assessed using the Shapiro–Wilk normality test. At 6 months, PLB and PTH were compared using *t* tests (A, B) or Mann–Whitney tests (C, D). At 30 months, CON, PTH, and PTHw were compared using one‐way ANOVA tests followed by Tukey's multiple comparison tests. Differences between PTH at 6 months and PTH and PTHw at 30 months were determined using one‐way ANOVA tests followed by Dunnett's multiple comparison tests (A–D) (A: *p* = 0.029, *F* = 4.5; B: *p* = 0.031, *F* = 4.4) or Kruskal–Wallis tests followed by Dunn's multiple comparison tests (C, D). *p* values <0.1 are noted.

The detailed histomorphometric analysis included 5971 intracortical pores (100 ± 49 pores/biopsy), which were classified according to their remodeling type and stage. After 6 months of treatment, the percentage of pores that were non‐quiescent (i.e., pores that were undergoing erosion and/or formation) was significantly (*p* < 0.001) higher in the PTH group [59 (46 to 68)%] than in the placebo group [41 (17 to 57)%] (Fig. 3A). After 30 months of treatment, the percentage of non‐quiescent pores remained significantly higher in the PTH group [71 (61 to 74)%] than in the CON group [52 (49 to 59)%, (*p* = 0.045)] and PTHw group [51 (45 to 63)%, (*p =* 0.043)] (Fig. 3A). The opposite was the case for quiescent pores (Fig. 3B). In general, the non‐quiescent pores (77% to 92%) contributed more to the total pore area than the quiescent pores (8.5% to 23%) (Fig. 3C,D, Table 1). After 6 months, the contribution of non‐quiescent pores to the total pore area was significantly higher in the PTH group [88 (81 to 95)%] than in the placebo group [77 (55 to 86)%, (*p <* 0.001)], while it did not differ between the groups after 30 months (Fig. 3C). Again, the opposite was the case for quiescent pores after 6 months (Fig. 3D). This aligns with the notion that PTH increases bone turnover not only in trabecular bone but also in cortical bone.

**Table 1:** Percentage of pores in different remodeling stages and divided according to their remodeling type, and their contribution to the total pore area

	6 months						30 months							
	Percentage of pore number			Percentage of pore area			Percentage of pore number				Percentage of pore area			
	PLB n = 21	PTH n = 21	p	PLB n = 21	PTH n = 21	p	CON n = 5	PTH n = 5	PTHw n = 8	Significance	CON n = 5	PTH n = 5	PTHw n = 8	Significance
Remodeling stages														
E	25 (11 to 51)	25 (9 to 37)^n^	0.31^m^	50 (27 to 73)^n^	22 (5 to 39)	**0.009** ^**m**^	46 (37 to 48)^n^	33 (32 to 37)^n^	43 (25 to 57)^n^	**e** ^**a**^	76 (69 to 85)^n^	45 (26 to 61)^n^	77 (56 to 84)^n^	**a** ^**a**^ **, b** ^**a**^ **, e** ^**k**^
EF	3.1 (1 to 7)	15 (9 to 19)^n^	**<0.001** ^**m**^	6.1 (0.1 to 18)	44 (35 to 59)^n^	**<0.001** ^**m**^	7.5 (4 to 11)^n^	25 (17 to 26)	7 (3 to 21)	**a** ^**k**^, b^k^	11 (4 to 18)^n^	44 (22 to 70)^n^	16 (3 to 23)^n^	**a** ^**a**^ **, b** ^**a**^ **, e** ^**a**^
F	3.1 (0.8 to 6)	15 (7 to 27)	**<0.001** ^**m**^	0.4 (0.1 to 2.4)	10 (2.2 to 25)	**<0.001** ^**m**^	1.5 (0.5 to 7)	9.6 (8 to 16)^n^	2.1 (0.4 to 3)^n^	**a** ^**k**^ **, b** ^**k**^ **, e** ^**k**^	0.02 (0 to 3.1)	1.4 (0.8 to 5)^n^	0.1 (0 to 1.0)	**e** ^**k**^
Non‐Q	41 (17 to 57)^n^	59 (46 to 68)^n^	**<0.001** ^**T**^	77 (55 to 86)	88 (81 to 95)	**<0.001** ^**m**^	52 (49 to 59)^n^	71 (61 to 74)^n^	51 (45 to 63)^n^	**a** ^**a**^ **, b** ^**a**^	90 (73 to 96)^n^	92 (85 to 99)^n^	91 (73 to 93)^n^	–
Q	59 (42 to 82)^n^	42 (31 to 54)^n^	**<0.001** ^**T**^	23 (13 to 43)	11 (5 to 19)	**0.001** ^**m**^	48 (40 to 49)^n^	29 (26 to 39)^n^	49 (37 to 54)^n^	a^a^ **, b** ^**a**^	10 (3.6 to 27)^n^	8.5 (1.3 to 15)^n^	9.6 (6.7 to 27)^n^	–
Type 1 remodeling														
% of type 1	31 (26 to 37)^n^	28 (24 to 37)^n^	0.68^T^	5.0 (3 to 15)	12 (4 to 18)^n^	0.193^m^	30 (24 to 36)^n^	25 (18 to 34)^n^	25 (21 to 29)^n^	–	4.0 (1.5 to 7)^n^	3.9 (3 to 5.4)^n^	3.1 (2 to 7.9)^n^	**d** ^**a**^ **, e** ^**a**^
Type 1 remodeling stages														
E	10 (3 to 15)^n^	6.1 (4 to 11)^n^	0.31^T^	31 (0.3 to 6)	2.4 (0.4 to 4)	0.69^m^	16 (9 to 19)^n^	9.6 (6 to 12)^n^	9 (6 to 14)^n^	–	1.2 (0.6 to 3)^n^	1.2(0.7 to 1.4)^n^	1.3 (0.7 to 1.7)	–
EF	0 (0 to 2.1)	2.3 (1.6 to 6)^n^	**0.001** ^**m**^	0 (0 to 0.7)	3.2 (0.6 to 8)	**<0.001** ^**m**^	0.7 (0 to 3.4)	3.2 (2.2 to 6)	2.3 (1 to 4.5)^n^	–	0 (0 to 1.2)	0.2 (0.1 to 3)^n^	0.1 (0 to 0.5)	**e** ^**k**^
F	1.0 (0 to 2.8)	2.2 (1 to 12)	**0.01** ^**m**^	0 (0 to 0.1)	0.4 (0 to 4)	**0.01** ^**m**^	1 (0 to 1.3)^n^	3.3 (1.5 to 5)^n^	0.5 (0 to 1.6)	**a** ^**k**^ **, b** ^**k**^ **, e** ^**k**^	0 (0 to 0.02)	0.1(0.1 to 0.2)^n^	0.01(0 to 0.05)	**a** ^**a**^ **, e** ^**k**^
Non‐Q	15 (5 to 20)^n^	17 (13 to 21)	0.087^m^	1.8 (1 to 9)	10 (3 to 17)^n^	**0.008** ^**m**^	19 (9 to 22)^n^	14 (12 to 22)^n^	13 (11 to 16)^n^	–	1.2 (0.6 to 4)	1.8 (1.2 to 4)^n^	1.7 (0.9 to 3)^n^	**e** ^**k**^
Q	18 (14 to 23)^n^	12 (7 to 15)^n^	**<0.001** ^**T**^	2.9 (1 to 5)	1.2 (0.5 to 2)	**0.009** ^**m**^	12 (10 to 17)^n^	9.4 (5 to 14)^n^	13 (8 to 15)^n^	–	1.6 (0.9 to 4)^n^	1.7 (0.3 to 3)^n^	1.6 (0.4 to 5)^n^	–
Type 2 remodeling														
% of type 2	69 (63 to 74)^n^	72 (63 to 76)^n^	0.68^T^	95 (85 to 97)	88 (82 to 96)^n^	0.19^m^	70 (64 to 76)^n^	75 (66 to 82)^n^	75 (71 to 79)^n^	–	96 (93 to 98)^n^	96 (95 to 97)^n^	97 (92 to 98)^n^	**d** ^**a**^ **, e** ^**a**^
Type 2 remodeling stages														
E	19 (7 to 33)^n^	16 (5 to 25)^n^	0.32^T^	53 (34 to 75)^n^	19 (2 to 37)	**<0.001** ^**m**^	29 (25 to 31)^n^	25 (21 to 29)^n^	34 (15 to 45)^n^	**e** ^**a**^	73 (50 to 83)^n^	43 (25 to 60)^n^	77 (55 to 88)^n^	b^a^, **e** ^**k**^
EF	1.6 (0 to 6)	11 (7 to 15)^n^	**<0.001** ^**m**^	5 (0 to 13)	41 (30 to 50)^n^	**<0.001** ^**m**^	6.7 (3.5 to 9)^n^	21 (13 to 22)^n^	2.8 (1.9 to 18)	**a** ^**k**^, b^k^	13 (3 to 25)^n^	44 (22 to 67)^n^	11 (0.5 to 21)^n^	**a** ^**a**^ **, b** ^**a**^ **, e** ^**a**^
F	1.7 (0 to 3.7)	13 (4 to 18)^n^	**<0.001** ^**m**^	0.2 (0 to 2.3)	8 (2 to 22)	**<0.001** ^**m**^	0 (0 to 6.5)	6.4 (5.7 to 12)	1.1 (0 to 2.1)^n^	**a** ^**k**^ **, b** ^**k**^ **, e** ^**k**^	0 (0 to 4.2)	1.3 (0.6 to 4)^n^	0.1 (0 to 1)	**e** ^**k**^
Non‐Q	27 (11 to 37)^n^	39 (32 to 49)^n^	**<0.001** ^**T**^	72 (50 to 84)	77 (66 to 90)^n^	0.14^m^	38 (32 to 42)^n^	55 (44 to 58)^n^	37 (34 to 51)	b^k^, **d** ^**k**^	88 (69 to 96)^n^	91 (84 to 94)^n^	89 (71 to 93)^n^	d^a^
Q	38 (30 to 55)^n^	26 (22 to 36)^n^	**0.004** ^**T**^	21 (11 to 36)	10 (3.6 to 16)	**0.003** ^**m**^	32 (29 to 35)^n^	22 (15 to 29)^n^	36 (23 to 40)^n^	a^a^, b^a^	8 (3 to 24)^n^	6.8 (1 to 12)^n^	8.1 (5.6 to 22)^n^	–

The treatment groups include 6 months with placebo (PLB) or rhPTH(1–84) (PTH) and 30 months with conventional treatment (CON) (6 months of placebo followed by conventional treatment for 24 months) and PTH or PTH withdrawal (PTHw) (6 months of PTH followed by conventional treatment for 24 months). The reported values are medians with interquartile ranges. Normality was tested using Shapiro–Wilk normality test: normally distributed data (^n^). At 6 months, PLB and PTH were compared using *t* tests (^T^) or Mann–Whitney test (m). At 30 months, CON, PTH, and PTHw were compared using one‐way ANOVA tests followed by Tukey's multiple comparison tests (^a^) or Kruskal–Wallis tests followed by Dunn's multiple comparison tests (^*k*^). Differences between PTH at 6 months and PTH and PTHw at 30 months were determined using one‐way ANOVA tests followed by Dunnett's multiple comparison tests (^a^) or Kruskal–Wallis tests followed by Dunn's multiple comparison tests (^k^). Differences between PTH at 6 months and PTH and PTHw at 30 months were determined using one‐way ANOVA tests followed by Dunnett's multiple comparison tests (^a^) or Kruskal–Wallis tests followed by Dunn's multiple comparison tests (^k^). (a) Significant difference between CON and PTH at 30 months, *p* ≤ 0.05 (bold) and *p* < 0.1 (not bold). (b) Significant difference between PTH and PTHw at 30 months, *p* ≤ 0.05 (bold) and *p* < 0.1 (not bold). (c) Significant difference between CON and PTHw at 30 months, *p* ≤ 0.05 (bold) and *p* < 0.1 (not bold). (d) Significant difference between PTH at 6 months and PTH at 30 months, *p* ≤ 0.05 (bold) and *p* < 0.1 (not bold). (e) Significant difference between PTH at 6 months and PTHw at 30 months, *p* ≤ 0.05 (bold) and *p* < 0.1 (not bold).

In general, type 2 pores (69% to 75%) were more prevalent than type 1 pores (25% to 31%) across all groups (Table 1). The contribution of type 2 pores to the total pore area was significantly higher in the PTH (*p =* 0.028) and PTHw (*p =* 0.026) groups after 30 months than in the PTH group after 6 months (Table 1). At 6 months, the percentage of quiescent type 1 and type 2 pores (*p <* 0.001 and *p =* 0.004) and their contribution to the pore area (*p =* 0.009 and *p =* 0.003) were significantly lower in the PTH group than in the PLB group (Table 1). No significant differences in quiescent type 1 and type 2 pores were observed after 30 months. The percentage of non‐quiescent type 2 pores was significantly higher in the PTH group than in the PLB group (*p <* 0.001) after 6 months, but their contribution to the pore area was not significantly higher (Table 1). On the other hand, the percentage of non‐quiescent type 1 pores was not significantly different between the PTH and PLB groups after 6 months, but their contribution to the pore area was significantly higher in the PTH group compared to the PLB group (*p =* 0.008). This suggests that PTH‐induced intracortical remodeling mainly remodels existing canals, rather than generating new canals.

### Effect of rhPTH(1–84) on magnitude of resorption and formation observed in quiescent osteons

**Fig. 4:**
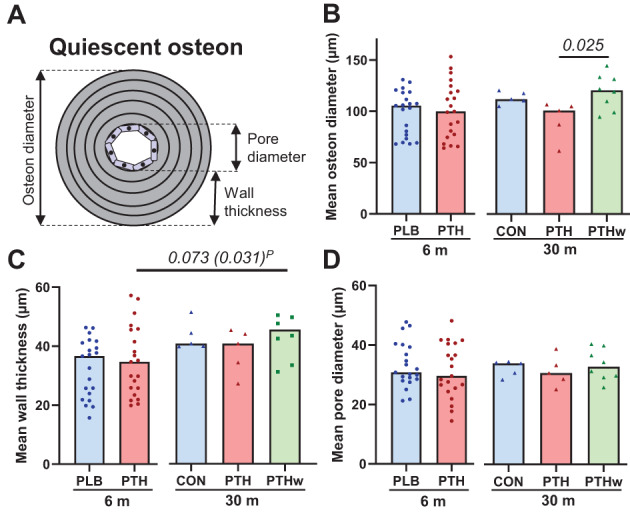
Effect of rhPTH(1–84) on magnitude of bone resorption and formation in quiescent osteons. (A) Schematic diagram of quiescent osteon illustrating different measurements, where osteon diameter reflects magnitude of bone resorption, wall thickness reflects magnitude of bone formation, and pore diameter reflects balance between the two. (B–D) The mean osteon diameter (B), mean wall thickness (C), and mean pore diameter (D) of quiescent osteons were not significant different between the PTH groups and PLB/CON groups, but the PTHw had slightly evelated mean osteon diameter and wall thickness in the respective treatment groups. Each dot represents a patient, and the bar represents the median. Normality was tested using the Shapiro–Wilk normality test. At 6 months, PLB and PTH were compared using *t* tests. At 30 months, CON, PTH, and PTHw were compared using one‐way ANOVA tests followed by Tukey's multiple comparison tests (B: *p* = 0.025, *F* = 4.8). Differences between PTH at 6 months and PTH and PTHw at 30 months were determined using one‐way ANOVA tests followed by Dunnett's multiple comparison tests and paired *t* test between the paired samples (P). *p* values <0.1 are noted.

The mean osteon diameter of the quiescent osteons was 105 ± 23 μm across groups and did not differ between PTH and PLB/CON treated groups after either 6 months or 30 months. However, it was significantly higher in the PTHw group compared to the PTH group after 30 months (Fig. 4B). Similarly, the mean wall thickness of the quiescent osteons across groups was 37 ± 11 μm and did not differ between PTH and PLB/CON treated groups after either 6 or 30 months, but it was significantly higher in the PTHw group than in the PTH group after 6 months (Fig. 4C). The mean pore diameter of quiescent osteons was 32 ± 7 μm across groups and did not differ between the PTH and PLB/CON treated groups after 6 or 30 months, as well as the PTHw group (Fig. 4D). Overall, this suggests that PTH has no effect on the magnitude of bone resorption (osteon diameter) or bone formation (wall thickness) but that PTH withdrawal results in a slightly larger magnitude of bone resorption and a compensatory larger magnitude of bone formation.

### Effect of rhPTH(1–84) on the remodeling stages of non‐quiescent pores

**Fig. 5:**
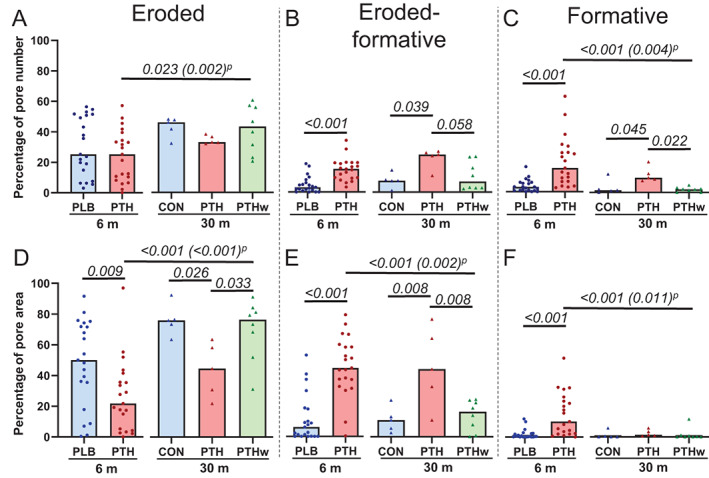
Effect of rhPTH(1–84) on eroded, eroded‐formative, and formative pores. Percentage of eroded (A), eroded‐formative (B), and formative (C) non‐quiescent cortical pores and their respective contributions to total pore area (D–F). The percentage of eroded pores was large unaffected, but the percentage of eroded‐formative and formative pores was significantly higher in the PTH groups (A–C). The percentage contribution of eroded pores to the total pore area was significantly lower in the PTH groups, while the contributions of eroded‐formative and formative pores were significantly higher (D–F). Each dot represents a patient, and the bar represents the median. Normality was tested using the Shapiro–Wilk normality test. At 6 months, PLB and PTH were compared using *t* tests (A) or Mann–Whitney tests (B–F). At 30 months, CON, PTH, and PTHw were compared using one‐way ANOVA tests followed by Tukey's multiple comparison tests (A, D, E) (D: *p* = 0.018, *F* = 5.3; E: *p* = 0.004, *F* = 8.0) or Kruskal–Wallis tests followed by Dunn's multiple comparison tests (B, C, F) (B: *p* = 0.035; C: *p* = 0.008). Differences between PTH at 6 months and PTH and PTHw at 30 months were determined using one‐way ANOVA tests followed by Dunnett's multiple comparison tests (A, E) (A: *p* = 0.032, *F* = 3.9; E: *p* = 0.001, *F* = 8.8) or Kruskal–Wallis tests followed by Dunn's multiple comparison tests (B–D, F) (B: *p* = 0.035; C: *p* < 0.001; D, F: *p* = 0.001), as well as by paired *t* test (P). *p* values <0.1 are noted.

The percentage of eroded pores (25% to 46%) did not differ between the PTH and PLB/CON treated groups after either 6 or 30 months (Fig. 5A, Table 1). However, the contribution of eroded pores to the total pore area was significantly lower in the PTH groups compared to the PLB/CON treated groups after 6 months (22 [5 to 39]% versus 50 [27 to 73]%) and 30 months (45 [26 to 61]% versus 76 [69 to 86]%) (Fig. 5D). On the other hand, the percentage of eroded pores and their contribution to the pore area were significantly higher in the PTHw group after 30 months than in the PTH group after 6 months and comparable to that of the CON treated group after 30 months (Fig. 5D).

Both the percentage of eroded‐formative and formative pores and their relative contribution to the total pore area were significantly higher in the PTH compared to the PLB group after 6 months (Fig. 5B–C,E–F). After 30 months, the percentage of eroded‐formative pores and their contribution to the total pore area remained significantly higher in the PTH group than in the CON treated group but were only borderline significantly larger than the PTHw group (Fig. 5B,E). Similarly, the percentage of formative pores remained significantly higher in the PTH group than in both the CON treated group and the PTHw group after 30 months (Fig. 5C). The contribution of formative pores to the total pore area was very low after 30 months and did not differ between the three groups (Fig. 5F). The percentage of formative pores and their contribution to the pore area was significantly lower in the PTHw group after 30 months than in the PTH group after 6 months (Fig. 5C,F).

When the pores were classified according to their remodeling type, the differences in eroded, eroded‐formative, and formative pores between the PTH and PLB groups after 6 months remained relatively similar (Table 1). Overall, the percentage of eroded pores did not differ between the PTH and PLB treated groups, but the percentages of eroded‐formative and formative pores in the PTH groups were significantly higher than in the PLB groups for both remodeling types (Table 1).

### Treatment with rhPTH(1–84) changes the ratio between eroded and formative pores

**Fig. 6:**
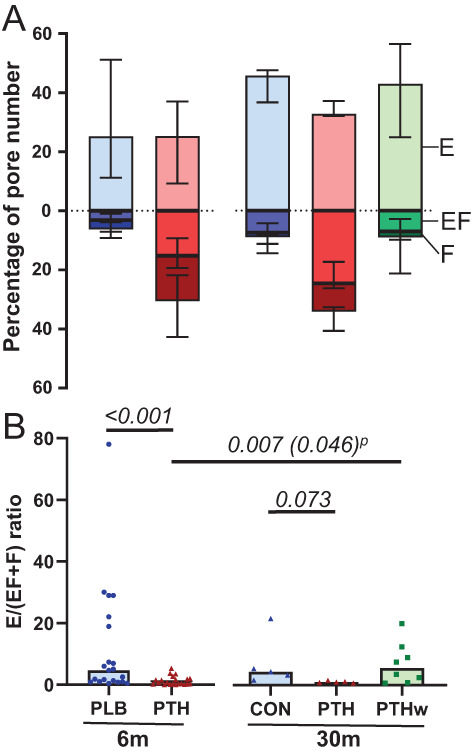
Effect of rhPTH(1–84) on ratio of eroded pores to eroded‐formative and formative pores. Ratio between eroded (E) and sum of eroded‐formative (EF) and formative (F) pores in respective treatment groups. The ratio was significantly lower in the PTH group compared to the PLB group at 6 months. (A) Bars represent median with interquartile range of percentage of E pores compared to EF and F pores, highlighting the shift in the pore remodeling stage between groups. (B) Each dot represents a patient, and bars represent the median. Normality was tested using the Shapiro–Wilk normality test. At 6 months, PLB and PTH were compared using Mann–Whitney tests. At 30 months, CON, PTH, and PTHw were compared using Kruskal–Wallis tests followed by Dunn's multiple comparison tests (*p* = 0.041). Differences between PTH at 6 months and PTH and PTHw at 30 months were determined using Kruskal–Wallis tests followed by Dunn's multiple comparison tests (*p* = 0.014), as well as by paired *t* test (P). *p* values <0.1 are noted.

After 6 and 30 months, the PTH groups had a higher percentage of eroded‐formative and formative pores compared to the PLB group after 6 months and the CON and PTHw groups after 30 months, resulting in a shift in the ratio of eroded pores to eroded‐formative and formative pores (Fig. 6A). The E/(EF + F) ratio was 4.7 (1 to 20) in the PLB group after 6 months and 4.4 (2.4 to 13) in the CON group after 30 months, reflecting an accumulation of eroded pores with a poor transition to formation (Fig. 6B). The E/(EF + F) ratio was sevenfold lower (*p <* 0.001) after 6 months and fourfold lower (*p =* 0.073) after 30 months in the PTH groups compared to the respective PLB/CON groups (Fig. 6B). The E/(EF + F) ratio of the PTHw group was comparable to that of the CON group after 30 months and significantly differed (*p =* 0.007) from that of the PTH group after 6 months (Fig. 6B). Overall, this suggests that PTH promotes the transition from eroded pores to formative pores, which is deficient in untreated hypoparathyroidism patients.

## Discussion

**Fig. 7:**
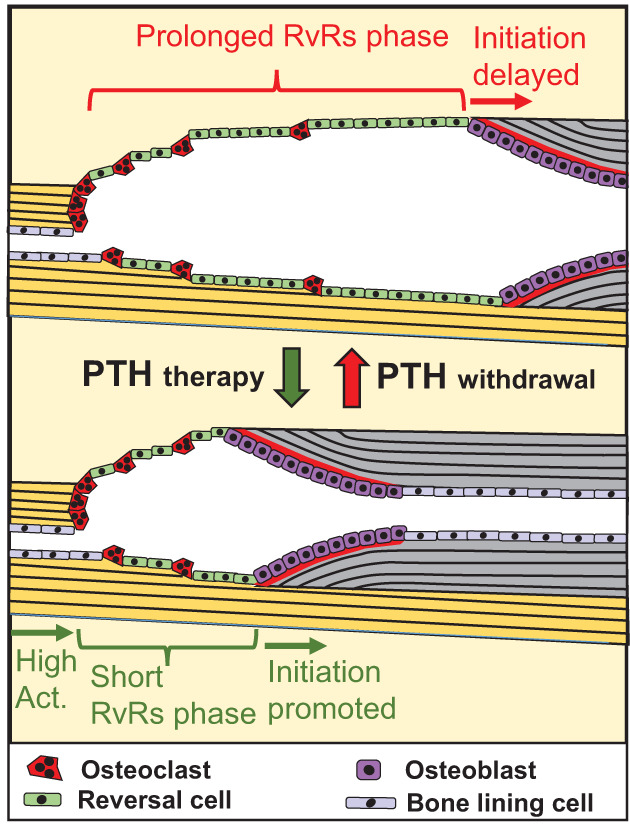
Model summarizing effect of intermittent rhPTH(1–84) on cortical bone remodeling in hypoparathyroidism patients. PTH treatment stimulated not only the activation of bone remodeling but also the transition from erosion to bone formation, shortening the reversal–resorption phase. Upon withdrawal of PTH treatment, bone remodeling resumed its original activation and delayed initiation of bone formation.

This study demonstrates that daily injections of rhPTH(1–84) into hypoparathyroidism patients reactivate intracortical bone remodeling and expedites the transition from bone erosion to formation, thereby reducing the duration of the reversal–resorption phase, as summarized in Fig. 7 and discussed in what follows. This reactivation of the intracortical bone remodeling persisted for the 30 months of the extension study but had no measureable effect on the overall cortical microstructure.

### rhPTH(1–84) treatment showed no effect on the cortical microstructure

Previous studies investigating the impact of intermittent PTH treatment on cortical porosity in patients with hypoparathyroidism or osteoporosis yielded conflicting results. Some studies showed that intermittent PTH‐treatment increased cortical porosity,^[^ ^9^, ^13^, ^16^, ^54^, ^55^, ^56^, ^57^, ^58^ ^]^ while other studies found no significant effect,^[^ ^14^, ^29^, ^52^ ^]^ as in the present study.

The discrepancy in study outcomes can be attributed to various factors:

- definition of border between cortical and trabecular bone, which influences cortical porosity assessment since cortical bone has a higher porosity in the endosteal region facing the trabecular compartment^[^ ^59^ ^]^;
- high intra‐individual variation, as observed in the present study;
- differences in underlying disease (osteoporosis versus hypoparathyroidism), cortical pathophysiology, and prior/combined treatment (naïve vs. bisphosphonate‐pretreated/combination)^[^ ^57^, ^60^ ^]^;
- skeletal site investigated;
- resolution of methodology employed;
- dosing and type of PTH (PTH[1–84] vs. PTH[1–34]).^[^ ^55^ ^]^

Furthermore, these factors may also account for the varying reports regarding the effect of PTH on cortical thickness, where some report an increased thickness,^[^ ^14^, ^29^, ^53^, ^55^, ^58^, ^60^ ^]^ while others report no significant effect or even a decrease in cortical thickness,^[^ ^30^, ^57^, ^60^, ^61^ ^]^ as in the present study.

Cortical porosity changes may result from alterations in pore density, pore size, or both. Even though our prior micro–computed tomography (μCT) study of the same biopsies reported an increased pore density in the rhPTH(1–84) versus PLB treated groups after 6 months,^[^ ^52^ ^]^ the present study observed no differences in pore density or size between the two groups. This discrepancy may be ascribed to the limited μCT resolution or the fact that the μCT analysis reported the canal diameter and the derived pore density volume‐weighted, which differs from the histological analyses conducted in the present study.

Overall, our study supports the notion that rhPTH(1–84) treatment of patients with hypoparathyroidism for either 6 or 30 months does not impact cortical microstructure.

### rhPTH(1–84) treatment stimulates intracortical bone remodeling and its transition to bone formation

This study establishes that intracortical bone remodeling in hypoparathyroidism patients is activated after 6 months of rhPTH(1–84) treatment and remains active throughout the study period. This finding supports the notion that bone remodeling within the intracortical envelope is activated upon PTH treatment,^[^ ^29^, ^35^, ^47^ ^]^ similar to the trabecular and endocortical envelopes.^[^ ^13^, ^14^, ^62^ ^]^

The PTH‐induced non‐quiescent pores primarily resulted from type 2 remodeling events (i.e., remodeling of existing canals), while the percentage of non‐quiescent pores generated by type 1 remodeling events (i.e., creation of new canals) remained unchanged. Type 2 remodeling was previously shown to become the most prominent intracortical remodeling with age,^[^ ^23^ ^]^ correlating with increased pore density. This could explain why the PTH‐activated non‐quiescent pores primarily remodel existing canals.

The most profound effect of rhPTH(1–84) treatment is the increased abundance of eroded‐formative and formative pores, reflecting a PTH‐induced initiation of bone formation. This effect persisted even after 30 months of treatment. This long‐term effect contrasts with previous studies, where the fraction of osteoid and mineralizing surfaces was increased after 3 or 12 months of treatment when compared to baseline, but not after 24 or 72 to 120 months of rhPTH(1–84) treatment in hypoparathyroidism patients.^[^ ^13^, ^16^ ^]^ Differences in the study design (baseline controlled study versus our open‐label extension study) and cortical histomorphometry methods (surface‐based versus our pore classification designed for cortical bone) may explain these discrepancies.

The activation of intracortical bone formation by PTH treatment may depend on the disease and type of PTH medication used. Studies conducted in osteoporosis and osteoarthritis patients have primarily focused on the impact of abaloparatide (a PTHrP analog)^[^ ^63^ ^]^ and PTH(1–34).^[^ ^36^, ^60^, ^64^ ^]^ Abaloparatide treatment increased the percentage of mineralizing surfaces after 3 months,^[^ ^63^ ^]^ while treatment with PTH(1–34) generally showed no significant effect on the fraction of osteoid or mineralizing surfaces after 1.5, 7 to 9, or 24 months.^[^ ^36^, ^60^, ^64^ ^]^ This suggests that rhPTH(1–84) exerts a unique effect in hypoparathyroidism patients when compared to the effect of PTH(1–34) and abaloparatide in osteoporosis and osteoarthritis patients.

rhPTH(1–84) treatment reduced the ratio of eroded pores to eroded‐formative and formative pores, which is even higher in hypoparathyroidism patients than in elderly and osteoporotic patients.^[^ ^23^, ^65^ ^]^ rhPTH(1–84) increases the proportion of remodeling pores by increasing the proportion of formative pores, while leaving the proportion of eroded pores unchanged. This leads to a high E/(EF + F) ratio, similar to that observed in younger individuals.^[^ ^23^ ^]^ This implies that the newly PTH‐activated remodeling events have a faster transition from bone erosion to formation. Thus, the E/(EF + F) ratio reflects the length of the reversal–resorption phase (eroded pores) relative to the length of the formation phase. Therefore, a high E/(EF + F) ratio reflects a prolonged reversal–resorption phase with a poor delayed transition from erosion to formation as observed in elderly, osteoporotic patients and hypoparathyroidism patients^[^ ^23^, ^65^ ^]^ (this study). In contrast, rhPTH(1–84) treatment appears to normalize this ratio in hypoparathyroidism patients, bringing it closer to the ratio observed in 20‐year‐old women (1.3 [95% CI 0.9 to 1.8]).^[^ ^23^ ^]^ On the other hand, the increased proportion of eroded‐formative and formative pores could also reflect the fact that the bone formation phase is longer upon rhPTH(1–84) treatment. However, this would require either an increased wall thickness increased or reduced mineral apposition rate. Notably, the wall thickness was unchanged upon rhPTH(1–84), and other studies reported that PTH(1–84) treatment resulted in an unaltered or even an increased mineral apposition rate.^[^ ^13^, ^16^, ^56^ ^]^

In previous studies, the proportion of eroded surface increased after 12 or 72 to 120 months of treatment, but not after 24 months of treatment.^[^ ^13^, ^16^ ^]^ In the present study, the percentage of eroded pores remained unaffected by rhPTH(1–84) treatment. The reduced E/(EF + F) ratio was mainly driven by an increased percentage of eroded‐formative and formative pores. Still, this does not imply that eroded pores are unaffected by rhPTH(1–84); rather, they have a faster transition to formation through a shorter reversal–resorption phase, coupled with an increased generation of new eroded pores.

The initiation of bone formation in eroded pores requires a critical density of osteoblastic reversal cells (osteoprogenitors).^[^ ^19^ ^]^ These osteoprogenitors are obtained through proliferation and recruitment from the pore lumen, where the osteoprogenitors ultimately originate from the vasculature.^[^ ^66^, ^67^ ^]^ Future studies are needed to clarify whether rhPTH(1–84) treatment induces osteoprogenitor proliferation and/or recruitment within intracortical remodeling events.

### rhPTH(1–84) treatment showed no effect on magnitude of bone resorption and formation

This study demonstrates that treatment of hypoparathyroidism patients with rhPTH(1–84) did not alter the final resorption depth (osteon diameter) before the initiation of bone formation, even though the eroded pores transitioned faster to formation upon rhPTH(1–84) treatment. In young healthy controls, the final resorption depth correlated with the length of the reversal–resorption phase in type 1 remodeling events,^[^ ^19^ ^]^ while the contribution of secondary resorption in type 2 remodeling events has yet to be investigated. Additionally, the relationship between final resorption depth and the length of the reversal–resorption phase found that type 1 remodeling events of healthy individuals^[^ ^19^ ^]^ may not apply to the accumulated eroded pores in hypoparathyroidism patients reactivated by rhPTH(1–84) treatment. The fact that the osteon diameter was not reduced when eroded pores transitioned faster to formation might suggest a relatively poor contribution of secondary resorption in rhPTH(1–84)‐treated patients with hypoparathyroidism. To our knowledge, no other study has investigated the effect of PTH treatment on the final resorption depth in cortical bone.

Wall thickness is a measure of the radial magnitude of the formed bone.^[^ ^68^ ^]^ In the present study, rhPTH(1–84) treatment had no effect on the magnitude of bone formed once initiated, as previous shown in PTH(1–34)‐treated osteoarthritis patients.^[^ ^36^ ^]^ This implies that PTH‐induced bone formation fully refills the resorption cavities. Accordingly, the balance between the radial magnitude of bone resorption and formation, which can be ascertained as the pore diameter of quiescent osteons, was not affected by the rhPTH(1–84) treatment.

### Effect of rhPTH(1–84) treatment withdrawal

In this study, a subgroup of patients received 6 months of treatment with rhPTH(1–84) and then crossed over to conventional treatment for 24 months. This allowed us to gain insights into the effect of withdrawing rhPTH(1–84) treatment. In the PTH withdrawal group, the skeletal condition of the patients fully reversed to that of the placebo/conventionally treated hypoparathyroidism patients. This means that the effects of the 6 months of rhPTH(1–84) treatment were completely reversed. These findings align with systemic studies of bone turnover markers, which have shown a gradual return to the baseline level after 3 to 6 months after discontinuing PTH treatment.^[^ ^37^, ^39^, ^42^, ^46^ ^]^ This further supports the conclusion that the effects of PTH are transitory.

## Limitations

The study has certain limitations, which we acknowledge. The extension of the study from 6 to 30 months involves an open‐label study design with a smaller number of patients, which increases the likelihood of encountering a type II error in the statistical analysis. Nevertheless, we were able to demonstrate that some effects of PTH were maintained until month 30, while other effects did not. It is important to note that the lack of statistical significance at month 30 could be attributed to the limited number of patients at this time point.

The relatively small number of patients in the 30‐month groups resulted in variations in patient age, gender, and etiology (Supplementary Table S1), which we acknowledge. Ideally, the study could have benefited from baseline biopsies; however, obtaining multiple biopsies from the same individual is challenging. While we successfully obtained paired biopsies from 6 and 30 months, it is worth noting that the pairing was not perfect, as some biopsies had to be excluded due to quality issues.

## Conclusion

This study showed that rhPTH(1–84) treatment of patients with hypoparathyroidism significantly increased intracortical bone turnover throughout the duration of the clinical study (30 months) but not the overall cortical microstructure. Importantly, this treatment promoted the transition of bone erosion to bone formation, thereby shortening the reversal–resorption phase, which had been prolonged in patients with hypoparathyroidism (this study), as well as in elderly^[^ ^23^ ^]^ and osteoporotic patients^[^ ^22^ ^]^ (Fig. 7).

These effects of rhPTH(1–84) were found to be transitory, as withdrawal of the treatment led to the normalization of bone remodeling to the levels of the PLB/CON group. Overall, this is the first study to demonstrate that PTH therapy in patients can directly rescue the cellular mechanisms previously reported to be responsible for bone loss in elderly and osteoporotic patients.

## Funding Information

The VELUX Foundation (VELUX25723), a Danish Southern Region Research Grant (18/17871), Aase and Ejnar Danielsen Foundation (10‐001584), Nycomed (for free study drugs (rhPTH[1–84] and similar placebo), Danish Council for Independent Research in Medical Science, The Novo Nordic Foundation, and the Central Denmark Region Foundation.

## Disclosures

Tanja Sikjær received an unrestricted research Grant from Shire (Takeda) to finance an unrelated study and received speaker's fees from Takeda and UCB. Lars Rejnmark received a research grant and is a research investigator and/or advisory board member of Takeda, Amolyt, Kyowa Kirin, Ascendis Pharma, and Calcilytix Therapeutics. Annemarie Brüel and Jesper Skovhus Thomsen received a research grant from Keros Therapeutics for an unrelated study. Thomas Levin Andersen received reagents for free and collaborated with ACD Bioscience, 10X Genomics, NanoString, ROWIAK LaserLabSolutions, Amgen, Merck, and Shire on unrelated studies.

## Author Contributions

**Pernille van Dijk Christiansen:** Data curation; formal analysis; funding acquisition; investigation; methodology; writing – original draft. **Tanja Sikjær:** Conceptualization; data curation; formal analysis; funding acquisition; investigation; methodology; resources; writing – review and editing. **Christina Møller Andreasen:** Conceptualization; data curation; formal analysis; funding acquisition; investigation; methodology; project administration; supervision; writing – review and editing. **Jesper Skovhus Thomsen:** Conceptualization; resources; validation; visualization; writing – review and editing. **Annemarie Brüel:** Formal analysis; supervision; writing – review and editing. **Ellen Margrethe Hauge:** Data curation; resources; writing – review and editing. **Jean‐Marie Delaisse:** Conceptualization; methodology; writing – review and editing. **Lars Rejnmark:** Conceptualization; data curation; formal analysis; funding acquisition; investigation; methodology; project administration; resources; supervision; writing – review and editing. **Thomas Levin Andersen:** Conceptualization; data curation; funding acquisition; investigation; methodology; project administration; resources; software; supervision; validation; visualization; writing – original draft; writing – review and editing.

### Peer Review

The peer review history for this article is available at https://www.webofscience.com/api/gateway/wos/peer-review/10.1002/jbm4.10829.

## Supporting information


**Supplementary Table S1.** Characteristics of Study Participant in the Respective Study Groups, Which Have No Significant Difference, Even Though the Patients in the 30 Months Groups Have Variable Etiologies.

## References

[1] Rosen CJ , Bilezikian JP . Clinical review 123: anabolic therapy for osteoporosis. J Clin Endocrinol Metab. 2001;86(3):957–964. 11238469 10.1210/jcem.86.3.7366

[2] Goltzman D . Physiology of parathyroid hormone. Endocrinol Metab Clin North Am. 2018;47(4):743–758. 30390810 10.1016/j.ecl.2018.07.003

[3] Silva BC , Bilezikian JP . Parathyroid hormone: anabolic and catabolic actions on the skeleton. Curr Opin Pharmacol. 2015;22:41–50. 25854704 10.1016/j.coph.2015.03.005 PMC5407089

[4] Wu M , Deng L , Zhu G , Li YP . G protein and its signaling pathway in bone development and disease. Front Biosci (Landmark Ed). 2010;15:957–985. 20515736 10.2741/3656

[5] Augustine M , Horwitz MJ . Parathyroid hormone and parathyroid hormone‐related protein analogs as therapies for osteoporosis. Curr Osteoporos Rep. 2013;11(4):400–406. 24078470 10.1007/s11914-013-0171-2 PMC3874264

[6] Shoback D . Clinical practice. Hypoparathyroidism. N Engl J Med. 2008;359(4):391–403. 18650515 10.1056/NEJMcp0803050

[7] Cusano NE , Rubin MR , Bilezikian JP . PTH(1‐84) replacement therapy for the treatment of hypoparathyroidism. Expert Rev Endocrinol Metab. 2015;10(1):5–13. 25705243 10.1586/17446651.2015.971755 PMC4334142

[8] Duan Y , De Luca V , Seeman E . Parathyroid hormone deficiency and excess: similar effects on trabecular bone but differing effects on cortical bone. J Clin Endocrinol Metab. 1999;84(2):718–722. 10022443 10.1210/jcem.84.2.5498

[9] Gafni RI , Brahim JS , Andreopoulou P , et al. Daily parathyroid hormone 1‐34 replacement therapy for hypoparathyroidism induces marked changes in bone turnover and structure. J Bone Miner Res. 2012;27(8):1811–1820. 22492501 10.1002/jbmr.1627 PMC3399961

[10] Agency EM . Natpar European medicines Agency: European Medicines Agency; 2017. [cited 2022 Oct 20]. Available from: https://www.ema.europa.eu/en/medicines/human/EPAR/natpar.

[11] Sikjaer T , Amstrup AK , Rolighed L , Kjaer SG , Mosekilde L , Rejnmark L . PTH(1‐84) replacement therapy in hypoparathyroidism: a randomized controlled trial on pharmacokinetic and dynamic effects after 6 months of treatment. J Bone Miner Res. 2013;28(10):2232–2243. 23649554 10.1002/jbmr.1964

[12] Silva BC , Bilezikian JP . Skeletal abnormalities in hypoparathyroidism and in primary hyperparathyroidism. Rev Endocr Metab Disord. 2020;22(4):789–802. 33200346 10.1007/s11154-020-09614-0

[13] Rubin MR , Dempster DW , Sliney J Jr , et al. PTH(1‐84) administration reverses abnormal bone‐remodeling dynamics and structure in hypoparathyroidism. J Bone Miner Res. 2011;26(11):2727–2736. 21735476 10.1002/jbmr.452 PMC4019384

[14] Hodsman AB , Kisiel M , Adachi JD , Fraher LJ , Watson PH . Histomorphometric evidence for increased bone turnover without change in cortical thickness or porosity after 2 years of cyclical hPTH(1‐34) therapy in women with severe osteoporosis. Bone. 2000;27(2):311–318. 10913928 10.1016/s8756-3282(00)00316-1

[15] Nishikawa A , Ishida T , Taketsuna M , Yoshiki F , Enomoto H . Safety and effectiveness of daily teriparatide in a prospective observational study in patients with osteoporosis at high risk of fracture in Japan: final report. Clin Interv Aging. 2016;11:913–925. 27462147 10.2147/CIA.S107285 PMC4939987

[16] Rubin MR , Zhou H , Cusano NE , et al. The effects of long‐term administration of rhPTH(1‐84) in hypoparathyroidism by Bone histomorphometry. J Bone Miner Res. 2018;33(11):1931–1939. 29972871 10.1002/jbmr.3543 PMC6546298

[17] Holzer G , von Skrbensky G , Holzer LA , Pichl W . Hip fractures and the contribution of cortical versus trabecular bone to femoral neck strength. J Bone Miner Res. 2009;24(3):468–474. 19016592 10.1359/jbmr.081108

[18] Spadaro JA , Werner FW , Brenner RA , Fortino MD , Fay LA , Edwards WT . Cortical and trabecular bone contribute strength to the osteopenic distal radius. J Orthop Res. 1994;12(2):211–218. 8164094 10.1002/jor.1100120210

[19] Lassen NE , Andersen TL , Ploen GG , et al. Coupling of Bone resorption and formation in real time: new knowledge gained from human haversian BMUs. J Bone Miner Res. 2017;32(7):1395–1405. 28177141 10.1002/jbmr.3091

[20] Kristensen HB , Andersen TL , Marcussen N , Rolighed L , Delaisse JM . Osteoblast recruitment routes in human cancellous bone remodeling. Am J Pathol. 2014;184(3):778–789. 24412092 10.1016/j.ajpath.2013.11.022

[21] Abdelgawad ME , Delaisse JM , Hinge M , et al. Early reversal cells in adult human bone remodeling: osteoblastic nature, catabolic functions and interactions with osteoclasts. Histochem Cell Biol. 2016;145(6):603–615. 26860863 10.1007/s00418-016-1414-y

[22] Andersen TL , Abdelgawad ME , Kristensen HB , et al. Understanding coupling between bone resorption and formation: are reversal cells the missing link? Am J Pathol. 2013;183(1):235–246. 23747107 10.1016/j.ajpath.2013.03.006

[23] Andreasen CM , Delaisse JM , van der Eerden BC , van Leeuwen JP , Ding M , Andersen TL . Understanding age‐induced cortical porosity in women: the accumulation and coalescence of eroded cavities upon existing intracortical canals is the Main contributor. J Bone Miner Res. 2018;33(4):606–620. 29193312 10.1002/jbmr.3354

[24] Dempster DW . Tethering formation to resorption: reversal revisited. J Bone Miner Res. 2017;32(7):1389–1390. 28498616 10.1002/jbmr.3169

[25] Delaisse JM , Andersen TL , Kristensen HB , Jensen PR , Andreasen CM , Søe K . Re‐thinking the bone remodeling cycle mechanism and the origin of bone loss. Bone. 2020;141:115628. 32919109 10.1016/j.bone.2020.115628

[26] Everts V , Delaisse JM , Korper W , et al. The bone lining cell: its role in cleaning Howship's lacunae and initiating bone formation. J Bone Miner Res. 2002;17(1):77–90. 11771672 10.1359/jbmr.2002.17.1.77

[27] Jensen PR , Andersen TL , Hauge EM , Bollerslev J , Delaissé JM . A joined role of canopy and reversal cells in bone remodeling–lessons from glucocorticoid‐induced osteoporosis. Bone. 2015;73:16–23. 25497571 10.1016/j.bone.2014.12.004

[28] Chau JF , Leong WF , Li B . Signaling pathways governing osteoblast proliferation, differentiation and function. Histol Histopathol. 2009;24(12):1593–1606. 19795357 10.14670/HH-24.1593

[29] Dempster DW , Cosman F , Kurland ES , et al. Effects of daily treatment with parathyroid hormone on bone microarchitecture and turnover in patients with osteoporosis: a paired biopsy study. J Bone Miner Res. 2001;16(10):1846–1853. 11585349 10.1359/jbmr.2001.16.10.1846

[30] Zanchetta JR , Bogado CE , Ferretti JL , et al. Effects of teriparatide [recombinant human parathyroid hormone (1‐34)] on cortical bone in postmenopausal women with osteoporosis. J Bone Miner Res. 2003;18(3):539–543. 12619939 10.1359/jbmr.2003.18.3.539

[31] Sikjaer T , Rejnmark L , Rolighed L , Heickendorff L , Mosekilde L . The effect of adding PTH(1‐84) to conventional treatment of hypoparathyroidism: a randomized, placebo‐controlled study. J Bone Miner Res. 2011;26(10):2358–2370. 21773992 10.1002/jbmr.470

[32] Boyce BF , Xing L . The RANKL/RANK/OPG pathway. Curr Osteoporos Rep. 2007;5(3):98–104. 17925190 10.1007/s11914-007-0024-y

[33] Tam CS , Heersche JN , Murray TM , Parsons JA . Parathyroid hormone stimulates the bone apposition rate independently of its resorptive action: differential effects of intermittent and continuous administration. Endocrinology. 1982;110(2):506–512. 7056211 10.1210/endo-110-2-506

[34] Frolik CA , Black EC , Cain RL , et al. Anabolic and catabolic bone effects of human parathyroid hormone (1‐34) are predicted by duration of hormone exposure. Bone. 2003;33(3):372–379. 13678779 10.1016/s8756-3282(03)00202-3

[35] Osagie‐Clouard L , Sanghani A , Coathup M , Briggs T , Bostrom M , Blunn G . Parathyroid hormone 1‐34 and skeletal anabolic action: the use of parathyroid hormone in bone formation. Bone Joint Res. 2017;6(1):14–21. 28062525 10.1302/2046-3758.61.BJR-2016-0085.R1 PMC5227055

[36] Cosman F , Dempster DW , Nieves JW , et al. Effect of teriparatide on Bone formation in the human femoral neck. J Clin Endocrinol Metab. 2016;101(4):1498–1505. 26900640 10.1210/jc.2015-3698 PMC4880158

[37] Hodsman AB , Fraher LJ , Watson PH , et al. A randomized controlled trial to compare the efficacy of cyclical parathyroid hormone versus cyclical parathyroid hormone and sequential calcitonin to improve bone mass in postmenopausal women with osteoporosis. J Clin Endocrinol Metab. 1997;82(2):620–628. 9024265 10.1210/jcem.82.2.3762

[38] Orwoll ES , Scheele WH , Paul S , et al. The effect of teriparatide [human parathyroid hormone (1‐34)] therapy on bone density in men with osteoporosis. J Bone Miner Res. 2003;18(1):9–17. 12510800 10.1359/jbmr.2003.18.1.9

[39] Finkelstein JS , Wyland JJ , Leder BZ , et al. Effects of teriparatide retreatment in osteoporotic men and women. J Clin Endocrinol Metab. 2009;94(7):2495–2501. 19401368 10.1210/jc.2009-0154 PMC2708954

[40] Finkelstein JS , Wyland JJ , Lee H , Neer RM . Effects of teriparatide, alendronate, or both in women with postmenopausal osteoporosis. J Clin Endocrinol Metab. 2010;95(4):1838–1845. 20164296 10.1210/jc.2009-1703 PMC2853981

[41] Leder BZ , Tsai JN , Uihlein AV , et al. Two years of denosumab and teriparatide administration in postmenopausal women with osteoporosis (the DATA extension study): a randomized controlled trial. J Clin Endocrinol Metab. 2014;99(5):1694–1700. 24517156 10.1210/jc.2013-4440 PMC4010689

[42] Lane NE , Sanchez S , Modin GW , Genant HK , Pierini E , Arnaud CD . Bone mass continues to increase at the hip after parathyroid hormone treatment is discontinued in glucocorticoid‐induced osteoporosis: results of a randomized controlled clinical trial. J Bone Miner Res. 2000;15(5):944–951. 10804025 10.1359/jbmr.2000.15.5.944

[43] Winer KK , Zhang B , Shrader JA , et al. Synthetic human parathyroid hormone 1‐34 replacement therapy: a randomized crossover trial comparing pump versus injections in the treatment of chronic hypoparathyroidism. J Clin Endocrinol Metab. 2012;97(2):391–399. 22090268 10.1210/jc.2011-1908 PMC3275355

[44] Winer KK , Ko CW , Reynolds JC , et al. Long‐term treatment of hypoparathyroidism: a randomized controlled study comparing parathyroid hormone‐(1‐34) versus calcitriol and calcium. J Clin Endocrinol Metab. 2003;88(9):4214–4220. 12970289 10.1210/jc.2002-021736

[45] Mannstadt M , Clarke BL , Bilezikian JP , et al. Safety and efficacy of 5 years of treatment with recombinant human parathyroid hormone in adults with hypoparathyroidism. J Clin Endocrinol Metab. 2019;104(11):5136–5147. 31369089 10.1210/jc.2019-01010 PMC6760337

[46] Glover SJ , Eastell R , McCloskey EV , et al. Rapid and robust response of biochemical markers of bone formation to teriparatide therapy. Bone. 2009;45(6):1053–1058. 19679211 10.1016/j.bone.2009.07.091

[47] Kurland ES , Cosman F , McMahon DJ , Rosen CJ , Lindsay R , Bilezikian JP . Parathyroid hormone as a therapy for idiopathic osteoporosis in men: effects on bone mineral density and bone markers. J Clin Endocrinol Metab. 2000;85(9):3069–3076. 10999788 10.1210/jcem.85.9.6818

[48] Winer KK , Sinaii N , Reynolds J , Peterson D , Dowdy K , Cutler GB Jr . Long‐term treatment of 12 children with chronic hypoparathyroidism: a randomized trial comparing synthetic human parathyroid hormone 1‐34 versus calcitriol and calcium. J Clin Endocrinol Metab. 2010;95(6):2680–2688. 20392870 10.1210/jc.2009-2464 PMC2902068

[49] Cusano NE , Rubin MR , McMahon DJ , et al. Therapy of hypoparathyroidism with PTH(1‐84): a prospective four‐year investigation of efficacy and safety. J Clin Endocrinol Metab. 2013;98(1):137–144. 23162103 10.1210/jc.2012-2984 PMC3537109

[50] Greenspan SL , Bone HG , Ettinger MP , et al. Effect of recombinant human parathyroid hormone (1‐84) on vertebral fracture and bone mineral density in postmenopausal women with osteoporosis: a randomized trial. Ann Intern Med. 2007;146(5):326–339. 17339618 10.7326/0003-4819-146-5-200703060-00005

[51] Oxlund H , Ejersted C , Andreassen TT , Torring O , Nilsson MH . Parathyroid hormone (1‐34) and (1‐84) stimulate cortical bone formation both from periosteum and endosteum. Calcif Tissue Int. 1993;53(6):394–399. 8293353

[52] Sikjaer T , Rejnmark L , Thomsen JS , et al. Changes in 3‐dimensional bone structure indices in hypoparathyroid patients treated with PTH(1‐84): a randomized controlled study. J Bone Miner Res. 2012;27(4):781–788. 22161686 10.1002/jbmr.1493

[53] Jiang Y , Zhao JJ , Mitlak BH , Wang O , Genant HK , Eriksen EF . Recombinant human parathyroid hormone (1‐34) [teriparatide] improves both cortical and cancellous bone structure. J Bone Miner Res. 2003;18(11):1932–1941. 14606504 10.1359/jbmr.2003.18.11.1932

[54] Macdonald HM , Nishiyama KK , Hanley DA , Boyd SK . Changes in trabecular and cortical bone microarchitecture at peripheral sites associated with 18 months of teriparatide therapy in postmenopausal women with osteoporosis. Osteoporos Int. 2011;22(1):357–362. 20458576 10.1007/s00198-010-1226-1

[55] Borggaard XG , Roux JP , Delaisse JM , Chavassieux P , Andreasen CM , Andersen TL . Alendronate prolongs the reversal‐resorption phase in human cortical bone remodeling. Bone. 2022;10(160):116419. 10.1016/j.bone.2022.116419 35413490

[56] Hansen S , Hauge EM , Beck Jensen JE , Brixen K . Differing effects of PTH 1‐34, PTH 1‐84, and zoledronic acid on bone microarchitecture and estimated strength in postmenopausal women with osteoporosis: an 18‐month open‐labeled observational study using HR‐pQCT. J Bone Miner Res. 2013;28(4):736–745. 23044908 10.1002/jbmr.1784

[57] Recker RR , Bare SP , Smith SY , et al. Cancellous and cortical bone architecture and turnover at the iliac crest of postmenopausal osteoporotic women treated with parathyroid hormone 1‐84. Bone. 2009;44(1):113–119. 18983947 10.1016/j.bone.2008.09.019

[58] Schafer AL , Burghardt AJ , Sellmeyer DE , et al. Postmenopausal women treated with combination parathyroid hormone (1‐84) and ibandronate demonstrate different microstructural changes at the radius vs. tibia: the PTH and ibandronate combination study (PICS). Osteoporos Int. 2013;24(10):2591–2601. 23589163 10.1007/s00198-013-2349-y

[59] Andreasen CM , Bakalova LP , Brüel A , et al. The generation of enlarged eroded pores upon existing intracortical canals is a major contributor to endocortical trabecularization. Bone. 2020;130:115127. 31689525 10.1016/j.bone.2019.115127

[60] Ma YL , Zeng QQ , Chiang AY , et al. Effects of teriparatide on cortical histomorphometric variables in postmenopausal women with or without prior alendronate treatment. Bone. 2014;59:139–147. 24269280 10.1016/j.bone.2013.11.011

[61] Nishiyama KK , Cohen A , Young P , et al. Teriparatide increases strength of the peripheral skeleton in premenopausal women with idiopathic osteoporosis: a pilot HR‐pQCT study. J Clin Endocrinol Metab. 2014;99(7):2418–2425. 24684466 10.1210/jc.2014-1041 PMC4079304

[62] Hodsman AB , Steer BM . Early histomorphometric changes in response to parathyroid hormone therapy in osteoporosis: evidence for de novo bone formation on quiescent cancellous surfaces. Bone. 1993;14(3):523–527. 8363903 10.1016/8756-3282(93)90190-l

[63] Dempster DW , Zhou H , Rao SD , et al. Early effects of Abaloparatide on Bone formation and resorption indices in postmenopausal women with osteoporosis. J Bone Miner Res. 2021;36(4):644–653. 33434314 10.1002/jbmr.4243 PMC8248188

[64] Dempster DW , Cosman F , Zhou H , Nieves JW , Bostrom M , Lindsay R . Effects of daily or cyclic teriparatide on Bone formation in the iliac crest in women on No prior therapy and in women on alendronate. J Bone Miner Res. 2016;31(8):1518–1526. 26916877 10.1002/jbmr.2822

[65] Borggaard XG , Nielsen MH , Delaisse JM , Andreasen CM , Andersen TL . Spatial organization of osteoclastic coupling factors and their receptors at human bone remodeling sites. Front Mol Biosci. 2022;9:896841. 35775083 10.3389/fmolb.2022.896841 PMC9239410

[66] Christiansen P , Andreasen CM , Laursen K , Delaisse JM , Andersen TL . Osteoprogenitor recruitment and differentiation during intracortical bone remodeling of adolescent humans 2022. 10.1016/j.bone.2023.116896 37699496

[67] Andreasen CM , El‐Masri BM , MacDonald B , et al. Local coordination between intracortical bone remodeling and vascular development in human juvenile bone. Bone. 2023;173:116787. 37150243 10.1016/j.bone.2023.116787

[68] Andreasen CM , Delaisse JM , van der Eerden BCJ , van Leeuwen J , Ding M , Andersen TL . Understanding age‐induced cortical porosity in women: is a negative BMU balance in quiescent osteons a major contributor? Bone. 2018;117:70–82. 30240959 10.1016/j.bone.2018.09.011

