# Supplementary material for: Transitory Activation and Improved Transition from Erosion to Formation within Intracortical Bone Remodeling in Hypoparathyroid Patients Treated with rhPTH(1–84)
Source: JBMR Plus. 2023 Nov 17;7(12):e10829. doi: 10.1002/jbm4.10829 (PMC10731115; doi:10.1002/jbm4.10829)
Supplement: Supplementary file 1 — Supplementary Table S1. Characteristics of Study Participant in the Respective Study Groups, Which Have No Significant Difference, Even Though the Patients in the 30 Months Groups Have Variable Etiologies. [file JBM4-7-e10829-s001.docx]

**Supplementary Table 1:** Characteristics of study participant in the respective study groups, which have no significant difference, even though the patients in the 30 months groups have variable etiologies .The treatment groups include 6 month treatment with placebo (PLB) or rhPTH(1–84) (PTH), and 30 months treatment with conventional treatment (CON) (6 months placebo followed by conventional treatment for 24 months), PTH or PTH withdrawal (PTHw) (6 months PTH followed by conventional treatment for 24 months). The reported values are medians with interquartile ranges. Normality was tested using Shapiro-Wilk normality test. At 6 months, PLB and PTH were compared using t-tests or Mann-Whitney test for age and disease duration, and chi-square test for gender and etiology. At 30 months, CON, PTH and PTHw were compared using one-way ANOVA tests followed by Tykey’s multiple comparison tests or Kruskal-Wallis tests followed by Dunn’s multiple comparison tests for age and disease duration, and chi-square test for gender and etiology.

|  |  | **6 months** | | | **30 months** | | | |
| --- | --- | --- | --- | --- | --- | --- | --- | --- |
|  | **ALL** | **PLB** | **PTH** | **p-value** | **CON** | **PTH** | **PTHw** | **p-value** |
| **Patients from which a biopsy was obtained** | **47**  **(5 only at 30 months)** | **21** | **21** |  | **5** | **5** | **8** |  |
| **Age** | **51 (31-74)** | **51 (37-73) years** | **53 (38-74) years** | **ns** | **43 (31-61 years)** | **49 (38-67) years** | **54 (40-74) years** | **ns** |
| **Genders** | **41 women**  **6 men** | **18 women**  **3 men** | **18 women**  **3 men** | **ns** | **5 women**  **0 men** | **5 women**  **0 men** | **7 women**  **1 man** | **ns** |
| **Disease duration** | **11 (1-37) years** | **10 (1-37) years** | **11 (2-29) years** | **ns** | **9 (2-14) years** | **7 (2-14) years** | **11 (2-29) years** | **ns** |
| **Surgical from**  **Atotic goiter** | **18 (39%)** | **8 (38%)** | **10 (48%)** | **ns** | **1 (20%)** | **4 (80%)** | **2 (25%)** | **ns** |
| **Surgical from**  **Toxic goiter** | **13 (28%)** | **7 (33%)** | **4 (19%)** | **ns** | **1 (20%)** | **0 (0%)** | **5 (63%)** | **ns** |
| **Surgical from**  **Thyroid cancer** | **8 (17%)** | **3 (14%)** | **5 (24%)** | **ns** | **1 (20%)** | **1 (20%)** | **0 (0%)** | **ns** |
| **Surgical from Primary hyperparathyroidism** | **3 (7%)** | **2 (10%)** | **1 (5%)** | **ns** | **0 (0%)** | **0 (0%)** | **1 (13%)** | **ns** |
| **Non-surgical (Idiopathic)** | **2 (9%)** | **1 (5%)** | **1 (5%)** | **ns** | **2 (40%)** | **0 (0%)** | **0 (0%)** | **ns** |
